# Mouse Lung and Spleen Natural Killer Cells Have Phenotypic and Functional Differences, in Part Influenced by Macrophages

**DOI:** 10.1371/journal.pone.0051230

**Published:** 2012-12-05

**Authors:** Tatiana Michel, Aurélie Poli, Olivia Domingues, Marion Mauffray, Maud Thérésine, Nicolaas H. C. Brons, François Hentges, Jacques Zimmer

**Affiliations:** 1 Laboratory of Immunogenetics and Allergology, Centre de Recherche Public de la Santé (CRP-Santé), Luxembourg, Luxembourg; 2 Core Facility Flow Cytometry, Centre de Recherche Public de la Santé (CRP-Santé), Luxembourg, Luxembourg; Beth Israel Deaconess Medical Center, Harvard Medical School, United States of America

## Abstract

NK cells are lymphocytes of the innate immune system which are a first line of defense against infections and tumor cells, in bone marrow and peripheral organs like lung and spleen. The lung is an organ in contact with respiratory pathogens and the site of inflammatory disorders triggered by the respiratory environment. In contrast, spleen is a lymphatic organ connected to the blood system which regulates the systemic immune response. Here we compare NK cell maturation and expansion as well as expression of NK cell receptors in spleen and lung compartments. We show that spleen and lung NK cells differ in phenotypic and functional characteristics due to a difference of maturity and cellular microenvironment. Indeed we observe that spleen and lung macrophages have the capacity to influence the cytotoxicity of NK cells by cell-to-cell contact. This suggests that the differences of NK cell subsets are in part due to a modulation by the organ environment.

## Introduction

NK cells are lymphocytes present all around the body, which contribute to trigger antiviral and anti-tumor defense. They participate in the resistance against infectious agents and influence the acquired immune response by cytotoxic activity and the secretion of cytokines, particularly IFN-γ but also Th2-associated cytokines such as IL-5 and IL-13 and the immunoregulatory cytokine IL-10 [1], [2]. NK cells are found in most organs, including bone marrow, spleen, lymph nodes, liver, lung and uterus [3], [4]. The tissue distribution of NK cells *in vivo* may impact their local role in immune responses. It has been described in the mouse, that NK cells, in various lymphoid or non-lymphoid organs, are quite different [5]–[8]. In bone marrow, NK cell subsets are considered precursors of a mature NK cell population, which is CD11b^high^ and predominantly found in peripheral organs such as the spleen, blood, liver and lung [9]. The lung is an important site of exposure to antigens and pathogens via its airways and its vascular system [10]. A viral infection will enhance NK cell activity in the lung or spleen, depending on whether the virus is given intranasally or intravenously. It was described that NK cells from spleen and lung have quite the same surface characteristics [11], [12], but their maturation is different, in fact there are more CD27^low^CD11b^high^ NK cells in the lung compared to the spleen [6], [12].

There is a considerable crosstalk between NK cells, dendritic cells (DCs) or macrophages. Several studies have shown that TLR-stimulated DCs or macrophages contribute to NK cell activation [13]–[20]. Macrophages are present in the environment of NK cells in the lung and in the spleen. It was shown that macrophages can influence NK cell activity. The crosstalk between human macrophages and NK cells activates the proliferation and the cytokine secretion of the latter, and primes NK cell cytotoxicity against potential target cells [16]. Furthermore, human alveolar macrophages are able to inhibit NK cell activity *in vitro* [17], [18]. In a rat model, it has been shown that NK cell activities are not inhibited by spleen macrophages [19]. Nevertheless, some major differences among species (rat, mouse and human) were found *in vitro* in the regulation of NK activity by macrophages [20].

In this study, we show in a mouse model that NK cells from the lung have other phenotypic and functional characteristics than NK cells from the spleen. Furthermore, we investigated the potentially different role of lung and spleen macrophages in the regulation of NK cell activity, by comparing *in vitro*, the NK cell cytotoxicity in the presence of these two subsets of macrophages. Results indicated that macrophages increased NK cell-mediated cytotoxicity against tumor cells. Interestingly, spleen macrophages have a tendency to prime further NK cells cytotoxicity than lung macrophages.

## Materials and Methods

### Mice

Female C57BL/6 mice were obtained from Harlan (The Netherlands). Mice were kept under specific pathogen-free conditions and were used at 6–10 weeks of age. All procedures respected current European regulations and were approved by the National Animal Research Authority.

### Cell Isolation and Culture

The mice were killed by cervical dislocation and spleen and lung were removed. Then cells were isolated using a digestion solution: collagenase II (1 ng/ml) (Invitrogen, Belgium), benzonase (50 U/ml) (Merck, Germany), MgCl2 (0.06 µM), 10% fetal bovine serum (Invitrogen) and treatment with red blood cell lysis buffer. Total spleen and lung cells were stained for FACS analysis, CFSE assay or incubated over-night with LPS (1 µg/ml), IL-12 (5 ng/ml) and IL-18 (100 ng/ml), or PMA (50 ng/ml)/ionomycin (1 µg/ml) and IL-4 (50 ng/ml) in complete medium : DMEM with 10% FCS, penicillin/streptomycin, 1 mM HEPES buffer and 50 µM 2-Mercaptoethanol supplemented with recombinant human IL-2 (1000 U/ml) (R&D Systems, UK). Macrophages were purified with the F4/80 biotin Ab and magnetic beads (anti-microbead Biotin isolation kit, Miltenyi Biotec, Belgium). NK cell purification was performed after a Nylon wool column (G.KISKER, Germany) as a negative selection, using magnetic beads and the F4/80 biotin Ab added in a cocktail of biotin-conjugated antibodies. (NK cell isolation kit II, Miltenyi Biotec).

Freshly isolated macrophages were incubated in 96 well plates or in transwells in 24 well plates. After 1 h, NK cells were incubated alone, in direct contact or in transwells with macrophages for 24 h at the ratio of 5∶1 in complete medium supplemented with IL-2.

### CFSE Assays

Spleen and lung cells were labeled with 10 µM of CFSE (Sigma-Aldrich, Belgium) for 15 min at 37°C in PBS with 0.2% BSA. After two washes, cells were resuspended in complete medium supplemented with IL-2 and put in culture for 4 days in 48 well plates. 7-AAD^−^ cells were gated for analysis.

### Degranulation and Cytokine Assays

Lysosomal granule exocytosis was determined by CD107a expression. After an over-night activation of spleen and lung cells at 37°C with different types of stimulating molecules (see cell isolation and culture part), cells were incubated for 5 minutes with anti-CD16/CD32 Ab (1 µg/10^6^ cells) followed by staining with anti-CD107a-FITC mouse-Ab or isotype control. After 1 h of incubation, Golgi stop (BD Biosciences, Belgium) was added for additional 4 h. Subsequently, intracellular staining for IL-5, IL-6, IL-10, IL-13, TNF-α, IFN-γ were performed.

### Cytotoxicity Assays

Cytotoxicity assays were performed as described previously [21]. Briefly NK cell cytotoxicity was determined after 24 h of incubation in 1000 U/ml of IL-2, or IL-2, IL-12 (5 ng/ml) and IL-18 (100 ng/ml), against the YAC-1 target cells that were labeled with 5 µM of CFSE. Effector cells (E) were mixed with target cells (T) at E/T ratios ranging from 1/1 to 25/1. After 4 h of incubation at 37°C, cells were analyzed on a FACSCanto flow cytometer. To identify dead cells, 15 µM of the dead cell marker TO-PRO-3 (Invitrogen, Belgium) was added. The percentage of dead cells during the test was normalized to the percentage of NK cells in lung and spleen suspensions. To assess NK cell cytotoxicity after coculture or transwell culture with macrophages, NK cells were removed by harvesting non-adherent cells and washing with medium. The purity of isolated NK cells was the same than the culture of NK cells alone (>90% NK 1.1^+^ CD3^−^ cells) as determined by flow cytometry.

### Flow Cytometry and Antibodies

Anti-mouse antibodies used for flow cytometry were as follows: CD107a-FITC, Ly49G2-FITC, 2B4-FITC, Ly49D-FITC, (BD Biosciences), Ly49H-FITC, CD27-FITC, Qa2-FITC, NKG2A/C/E-FITC (eBioscience, USA); Ly49A-PE, Ly49C/I-PE, Ly49F-PE NKG2D-PE (BD Biosciences), CD69-PE (Biolegend), IL-5-PE, IL-6-PE, IL-10-PE, IL-13-PE, TNF-α-PE, IFN-γ-PE, CD122-PE (eBioscience); NK1.1-PECy7, CD11b-APC, NKp46-APC and CD3-APC-Alexa780, KLRG1-biotin (eBioscience) and F4/80-biotin (Biolegend), Fluorescein (DTAF)-conjugated streptavidin (Jackson ImmunoResearch, UK). The viability was confirmed by staining the cells with live/dead fixable near-IR dead cells stain (Invitrogen). Cells were analyzed on a FACSCanto flow cytometer and the graphs were performed with Flow Jo software (Tree Star).

### Statistical Analysis

The statistical significance of differences was determined by an unpaired t test and Mann Whitney test using GraphPad software. The values were expressed as means ± SEM from independent experiments. Values of *p<0.05 were considered significant.

## Results and Discussion

### NK Cell Maturation and Receptor Expression Change Depending on the Organ

**Figure 1:**
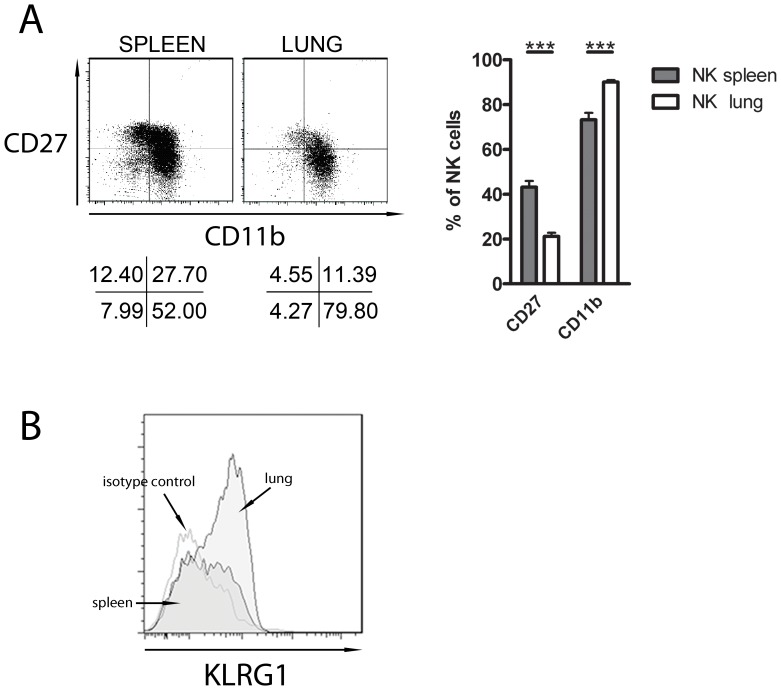
Differences of the mature phenotype between spleen and lung NK cells. NK cells (NK1.1^+^CD3^−^) from lung and spleen were analyzed by flow cytometry. (A) NK cells were analyzed for CD27 and CD11b expression. The data shown represent one out of seven experiments. Results for each group (n = 7) are expressed as means ± SEM. ***p<0.001 (Student’s unpaired t-test). (B) NK cells were gated for KLRG1 expression. The data shown represents one out of three experiments.

The ratio of the NK cell subsets differ between various compartments of the body. The most immature subset is CD27^high^CD11b^low^, followed by CD27^high^CD11b^high^ and CD27^low^CD11b^high^ subsets [9]. It has been described that the ratio of NK cell subsets varies between spleen and lung [6]. Here we confirm that the percentage of CD27^ low^CD11b^high^ NK cells is higher in the lung than in the spleen (Fig. 1, A). Lung NK cells expressed more CD11b than spleen NK cells (90,07% ±0,8 *versus* 73,24% ±3,1) and less CD27 (21,17% ±1,7 *versus* 43,20%±2,7) (n = 7) (Fig. 1, A).

KLRG1 is another marker of NK cell maturation, indeed it was shown that its expression at the NK cell surface characterizes the most mature NK cell subset [22], [23]. So we analyzed the presence of this receptor at the surface of spleen and lung CD3^−^NK1.1^+^ NK cells. We found that lung NK cells expressed more KLRG1 than spleen NK cells (61.9% *versus* 36.5%) (Fig. 1, B), confirming that lung NK cells have a more mature phenotype.

**Figure 2:**
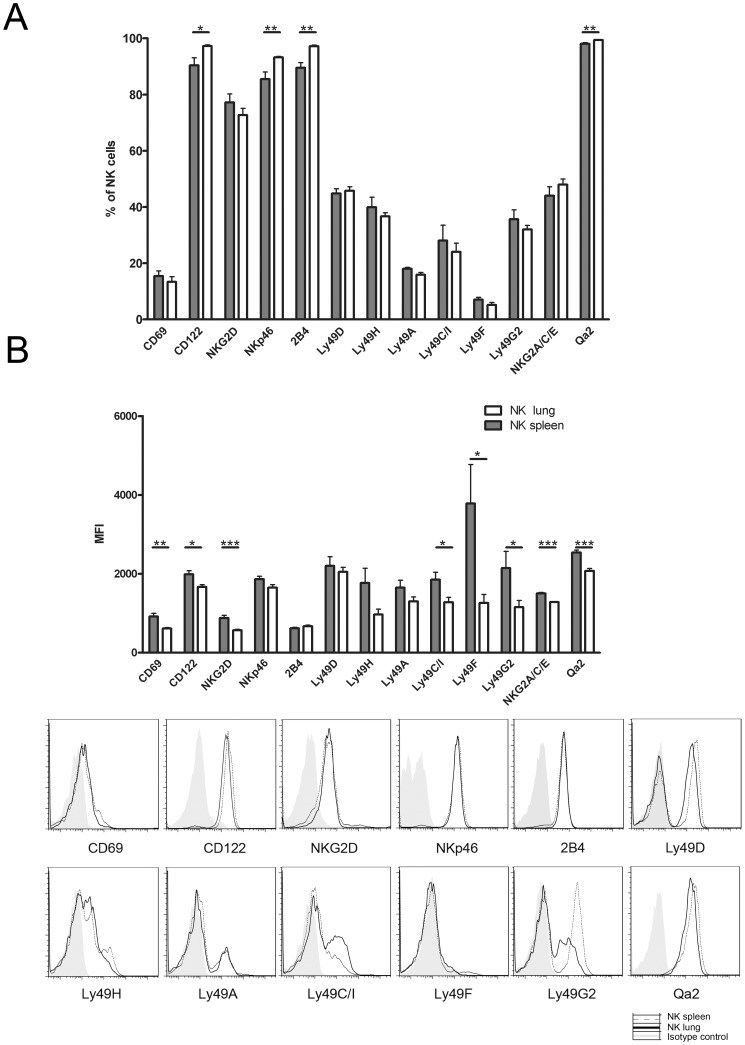
The level of receptor expression differs between spleen and lung NK cells. Spleen and lung cells were stained for NK cells (NK1.1^+^CD3^−^) and analyzed for several markers and Ly49 receptors by flow cytometry. (A) % of NK cells expressing the receptors. (B) MFI values of NK cell receptors. Results for each group (n = 7) are expressed as means ± SEM. *p<0.05, **p<0.01, ***p<0.001 (Student’s unpaired t-test).

CD3^−^NK1.1^+^ NK cells from spleen and lung were characterized for their receptor distribution. The frequency of NK cells which express CD122, NKp46, 2B4 and Qa2 is higher in lung NK cells compared to spleen NK cells. The CD27^low^CD11b^high^ subset of NK cells represents a terminally mature population with high expression of Ly49 receptors [6], [23]. The percentage of NK cells expressing the Ly49 receptor repertoire doesn’t change between these two organs (Fig. 2, A). These results are in accordance with data reported by Guo and Topham [12]. Surprisingly the expression level at the cell surface, reflected by the mean fluorescence intensities (MFI) of the Ly49 inhibitory receptors (Ly49C/I, Ly49F and G2) is higher in NK cells from the spleen than from the lung (Fig. 2, B). The MFI of CD69, CD122, NKG2D, NKG2A/C/E and Qa2 is also significantly higher in spleen NK cells (Fig. 2, B). These data indicated that lung NK cells have a more mature phenotype and expressed less receptors at their surface. Hayakawa et al., have found that CD27^low^CD11b^high^ NK cells displayed a skewed expression of inhibitory Ly49 receptors (C and I isoforms) [6]. Our results support the hypothesis that the more NK cells are CD27^low^CD11b^high^, the more this population is at the end stage of differentiation [9]. At this stage of maturation, lung NK cells may have a decrease of the capacity to present receptors at their surface [24]. Previous reports shown that CD3^−^ NKp46^+^ NK cells from the lung have more expression at their surface of Ly49D and Ly49C/I than those of the spleen [12], [25]. This contradiction with our results could be due to the fact that the gender of mice is different, the conditions of gating (CD3^−^NKp46^+^ population and not CD3^+^NK1.1^+^), the methods (digestion step) and the group of cells analyzed (purified lymphocytes and not total cells) are not the same.

### NK Cell Proliferation Changes Depending on the Organ

**Figure 3:**
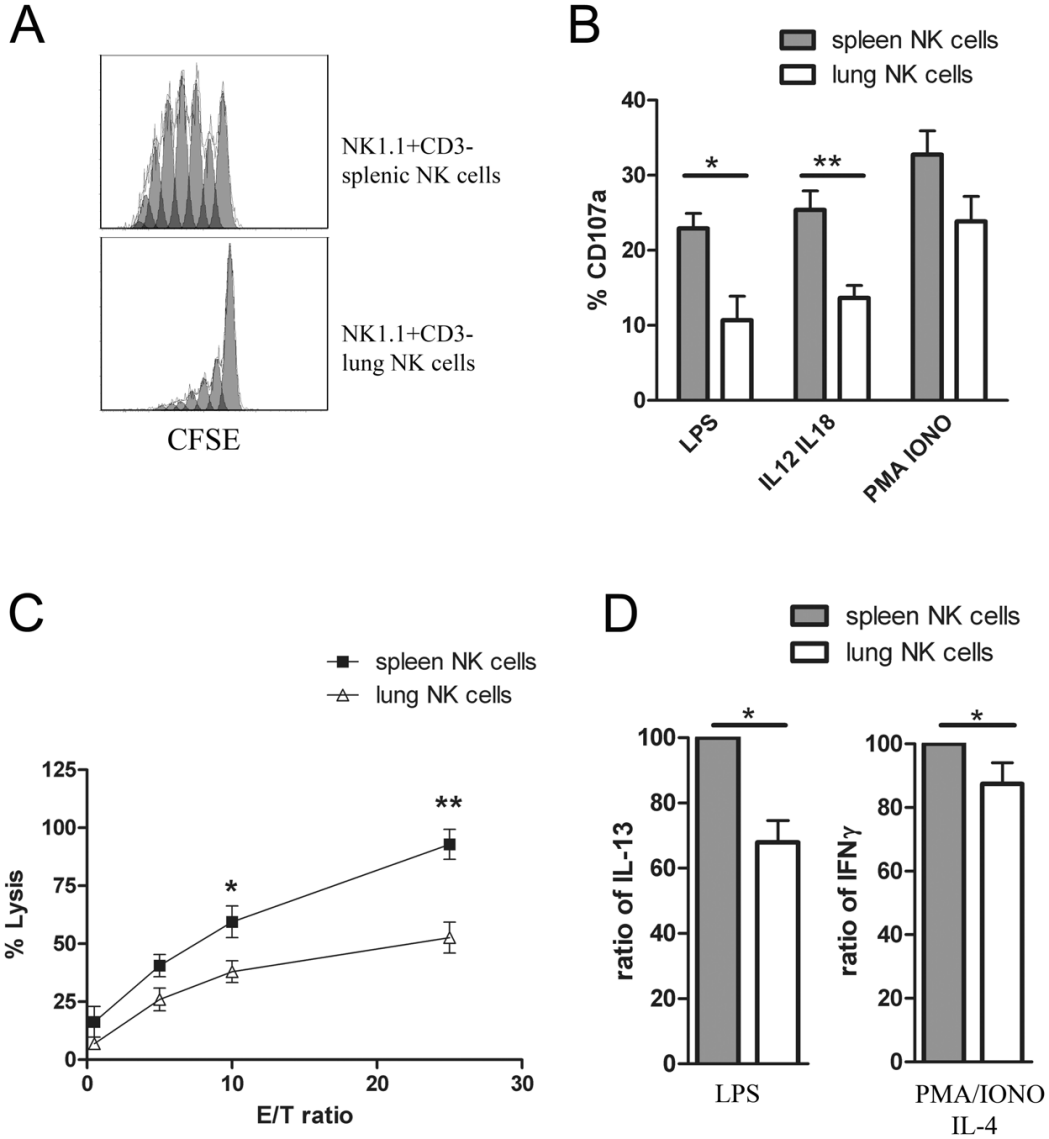
Spleen and lung NK cells have different proliferative and cytotoxic capacity. (A) Proliferation of 7AAD^−^ NK cells. Splenocytes and lung cells were stained with CFSE and analyzed by flow cytometry after 4 days in culture. From high to low the peaks are showing an additional cell cycle, the highest arbitrary fluorescence unit peak could be cells which didn’t cycle at all. Data are representative of three experiments. (B) Degranulation assay. Spleen and lung NK cells were stimulated with LPS, IL-12/IL-18 or PMA/ionomycin (PMA/IONO). CD3^−^NK1.1^+^ NK cells were analyzed for CD107a expression (n = 4). (C) Cytotoxicity assay was performed against YAC-1 target cells (n = 4). The results for each group are expressed as means ± SEM. *p<0.05, **p<0.01. (D) Cytokine levels. IL-13 and IFN-γ were produced by NK cells from the lung and the spleen after stimulation under different conditions: LPS or PMA/ionomycin/IL-4. Results are presented as a ratio of spleen NK cells (100%) to lung NK cells (n = 4). The results for each group are expressed as means ± SEM. *p<0.05.

In order to investigate if NK cells from the spleen and the lung have a different capacity to divide, we determined the proliferation rate of these two types of NK cells after CFSE staining. After 4 days in culture with IL-2, spleen NK cells displayed a stronger proliferative response than lung NK cells, as shown by the division profile in Fig. 3, A. We have found that lung NK cells express less CD122, the IL-2Rβ chain, at their surface. This may explain why these cells have a limited capacity to proliferate in comparison to spleen NK cells. This result is in accordance with previous data showing that the CD27^low^ NK cell subset has less proliferative potential [6], [9].

### NK Cell Degranulation and Cytotoxicity Change Depending on the Organ

We have compared the cytotoxic ability of lung NK cells and spleen NK cells. In three different conditions of activation (LPS, IL-12/IL-18 or PMA/ionomycin), spleen NK cells show a significantly stronger degranulation than lung NK cells (Fig. 3, B). This result correlates with the level of expression of the activating receptor CD69 found in lung and spleen NK cells, respectively. Furthermore the cytotoxic activity of spleen NK cells against YAC-1 target cells is two times higher than that of lung NK cells (92.8% ±6.5 *versus* 52.65% ±6.6) for the ratio 25/1 and the difference is also significant for the ratio 10/1 (Fig. 3, C). As described in a previous report, we found that the more the organ contains CD27^low^ NK cells, the less there is cytotoxic activity [6]. Under steady-state conditions, lung is continuously exposed to antigen and pathogen compared to spleen. Thus lung develops specific strategy to prevent local inflammation. In regard of our functional assay, we suggest that lung NK cells have less capacity to react against microorganisms compared to spleen NK cells in absence of infection. The regulation of their activities could result from the cell environment. Therefore, the tissue distribution of NK cells seems to condition their role in immune response.

### Cytokine Secretion Levels from NK Cells Change Depending on the Organ

Cytokine production by spleen and lung NK cells was determined by intracellular staining. Upon stimulation with LPS, or PMA/ionomycin during 12 h, the expression levels of IL-13 and IFN-γ were higher in spleen NK cells in comparison to lung NK cells (Fig. 3, D). The levels of IL-5, IL-6, IL-10 and TNF-α tended to be higher in spleen NK cells in comparison to lung NK cells but without reaching statistical significance (data not shown). It has been described that the CD27 receptor mediates IFN-γ production and that the CD27^high^CD11b^high^ NK cell subset shows the highest IFN-γ producing capacity [6], [26]. As the spleen has a higher percentage of this NK cell subset when compared to the lung, rationally we found that spleen NK cells have a higher cytokine production capacity. Studies have shown after following the trafficking of CFSE labeled splenocytes in mouse, that spleen NK cells are not programmed to home to the spleen but could be found in all NK cell-containing organs [4], [9], [27]. This suggests that differences found between spleen and lung NK cells could be due to the different environment. As presented here, the organ microenvironment seems to have a significant role in influencing cytokine production by NK cells. Therefore we tried to define the environmental impact on spleen and lung NK cell function.

### Influence of Macrophages on NK Cells

**Figure 4:**
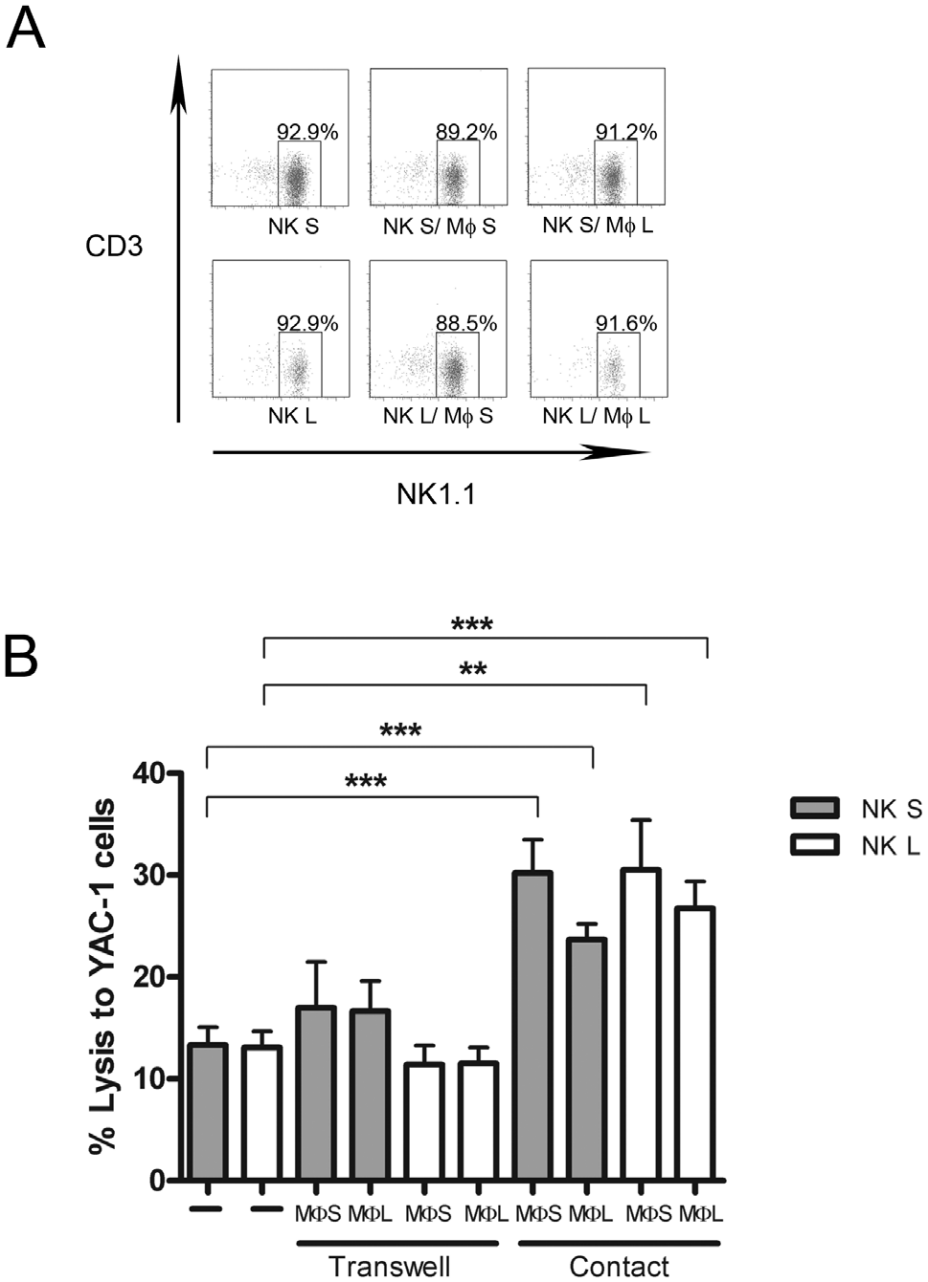
Influence of macrophages on NK cell cytotoxicity. (A) Purity of NK cells used for the cytotoxicity test after contact with macrophages. The data shown represents one out of three experiments. (B) Cytotoxicity assays were determined against YAC-1 target cells at E:T ratio 1∶2, after cell-to-cell contact or in transwells with macrophages or alone (n = 3). The results for each group are expressed as means ± SEM. **p<0.01, ***p<0.001. MФ: macrophage, NK S: NK spleen, NK L: NK lung.

It has been shown that human macrophages have the capacity to increase the activity of NK cells [16]. To observe the influence of macrophages on NK cell function in the mouse, lung and spleen macrophages and NK cells were purified and put in direct contact or separately in transwells. Then the cytotoxic capacity of NK cells was tested. Lung and spleen NK cell purity was the same before and after contact with macrophages (Fig. 4A). After cell-to-cell contact with macrophages, spleen and lung NK cells significantly increased their capacity to kill YAC-1 tumor cells (Fig. 4B). Spleen and lung macrophages are able to prime the cytotoxicity of NK cells, however they need to be in contact since the transwell conditions didn’t show any differences with the NK cells alone. Furthermore, the activity of NK cells seems to be higher by the interaction with spleen macrophages compared to lung macrophages (Fig. 4B). This could explain previous results showing that spleen NK cells are more cytotoxic than lung NK cells when the test is performed with total cells. In addition, we have analyzed and compared the level of IL-12, TNF-α and IFN-γ cytokine secretion in the supernatant of the different conditions of co-culture of macrophages and NK cells. Results have shown no differences in the presence or the absence of macrophages, which means that this interaction didn’t activate or inhibit the secretion of IL-12, TNF-α and IFN-γ (data not shown).

We have observed that lung NK cells have characteristics of more mature cells when compared with spleen NK cells. The level of the receptors present at the NK cell surface is lower in the lung and the functional capacities are also different between lung and spleen NK cells. Due to the NK cell subset distribution, we expected to have more activity in lung NK cells, yet we found less degranulation, cytotoxicity and cytokine production in this NK cell subset. The fact that the tissue-specific environment may influence the NK cell differentiation should be considered in view of our results showing the activating role of macrophages on NK cells function. Furthermore the cytokines IL-2, IL-12 and IL-15 influence the division and activation of NK cells. It was previously shown that IL-2, IL-12 and IL-15 mRNA are constitutively more expressed in spleen than lung tissues [28], [29]. Moreover IL-15 is predominantly expressed by adherent cells in spleen and lung, which contain a high proportion of macrophages/monocytes. In addition, IL-15 mRNA is also more expressed in spleen as compared to lung macrophages [29]. Finally, IL-15 was described to be secreted by alveolar macrophages in the mouse [30]. Other candidates like DC may regulate the NK cell function in spleen and lung, it was shown that local production of IL-15 by DC is required for the maintenance of the NK-cell compartment [31]. Further experiments with lung and spleen NK cells in presence of different cell partners need to be performed to better understand the differences found in NK cells from these two organs.
