# Correction: Mouse Lung and Spleen Natural Killer Cells Have Phenotypic and Functional Differences, in Part Influenced by Macrophages

**DOI:** 10.1371/annotation/93791c32-5d2e-4fbc-bd74-ba219c1bd79a

**Published:** 2013-01-15

**Authors:** Tatiana Michel, Aurélie Poli, Olivia Domingues, Marion Mauffray, Maud Thérésine, Nicolaas H. C. Brons, François Hentges, Jacques Zimmer

There was an error in the published Competing interests statement. The correct statement is: "Co-author Jacques Zimmer is a PLOS ONE Editorial Board member. We confirm that this does not alter our adherence to all the PLOS ONE polices on sharing data and materials." 

